# Peptides mediating DNA transport on microtubules and their impact on non-viral gene transfer efficiency

**DOI:** 10.1042/BSR20170995

**Published:** 2017-10-17

**Authors:** Patrick Midoux, Lucie Pigeon, Cristine Gonçalves, Chantal Pichon

**Affiliations:** Centre de Biophysique Moléculaire, CNRS UPR4301 and University of Orléans, Orléans 45071, cedex 02, France

**Keywords:** dynein, FIP-1, gene transfer, intracellular transport, microtubules, non-viral vector

## Abstract

Synthetic vectors such as cationic polymers and cationic lipids remain attractive tools for non-viral gene transfer which is a complex process whose effectiveness relies on the ability to deliver a plasmid DNA (pDNA) into the nucleus of non-dividing cells. Once in the cytosol, the transport of pDNAs towards the nuclear envelope is strongly impaired by their very low cytosolic mobility due to their large size. To promote their movement towards the cell nucleus, few strategies have been implemented to exploit dynein, the microtubule’s (MT’s) motor protein, for propagation of cytosolic pDNA along the MTs towards the cell nucleus. In the first part of this review, an overview on MTs, dynein, dynein/virus interaction feature is presented followed by a summary of the results obtained by exploitation of LC8 and TCTEL1 dynein light chain association sequence (DLC-AS) for non-viral transfection. The second part dedicated to the adenoviral protein E3-14.7K, reports the transfection efficiency of polyplexes and lipoplexes containing the E3-14.7K-derived P79-98 peptide linked to pDNA. Here, several lines of evidence are given showing that dynein can be targeted to improve cytosolic pDNA mobility and accumulate pDNA near nuclear envelope in order to facilitate its transport through the nuclear pores. The linkage of various DLC-AS to pDNA carriers led to modest transfection improvements and their direct interaction with MTs was not demonstrated. In contrast, pDNA linked to the P79-98 peptide interacting with TCTEL1 via a cytosolic protein (fourteen seven K-interacting protein-1 (FIP-1)), interaction with MTs is evidenced *in cellulo* and transfection efficiency is improved.

## Introduction

**Figure 1:**
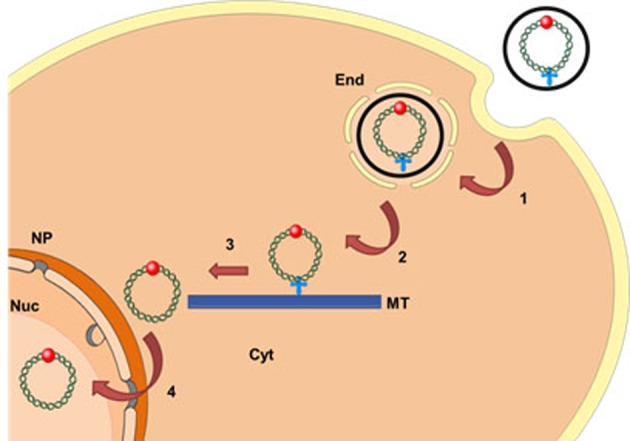
Scheme of the nuclear delivery of pDNA upon transfection with synthetic vectors ○, Synthetic vector; 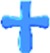, MT localization signal; 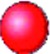, NLS; End, endosomes; Nuc, nucleus; NP, nuclear pore; Cyt, cytosol; MT, microtubule. Endocytosis (1); endosome escape (2); MT migration (3); nuclear import (4).

The relative non-immunogenicity and non-toxic nature of the synthetic vectors as well as their potential of targeting specific cells are increasingly making them the carriers of choice for DNA delivery and gene therapy. These vectors are based on cationic lipids, polymers or combination of lipid/polymer forming electrostatic complexes with plasmid DNA (pDNA). But to date, they have experienced inferior transfection efficiency compared with the viral vectors [1–4]. Synthetic vectors should be able to provide protection to the nucleic acid payload and promote the endosomal escape, unpacking of complex and release the nucleic acid in the cytosol (Figure 1). Furthermore, non-viral gene transfer is a complex process whose effectiveness relies on the ability of the therapeutic DNA to reach the nucleus. However, after internalization by endocytosis and delivery in the cytosol, the therapeutic DNA is highly vulnerable to intracellular DNases when dissociated from the carrier, and its delivery in the nucleus of non-dividing cells must pass through the nuclear pore of 30 nm in diameter. Indeed, the lifetime of pDNA in the cytosol has been reported to be in the range of 60–90 min in HeLa and COS-1 cells [5] and even shorter in muscle cells (~5 min) [6]. Moreover, its mobility is strongly reduced. Measurements by FRAP of the diffusion constants of nucleic acids after microinjection in the cytoplasm of HeLa cells showed that beyond 2000 bp, the diffusion of pDNA is very low or even zero for larger pDNA in the cytoplasm [7]. Several parameters can explain this reduced mobility in eukaryotic cells such as restrictive space and interaction of pDNA with cytosolic proteins notably with positively charged proteins. The cytoskeleton, in particular, the actin network appears to be the main obstacle to the diffusion of pDNA. After destabilization of the actin network, the mobile fraction of the pDNA and its diffusion constant were indeed increased [8]. Microinjection and electroporation experiments with naked pDNA, lead to the hypothesis that it can move into the cytosol along microtubules (MTs) by exploiting dynein, the MTs motor protein which exerts a movement towards the cell nucleus, when a DTS (DNA nuclear targeting sequence) is associated with pDNA [9]. For instance, it has been shown that the presence of five κB repeated units in tandem (5′-GGGGACTTTCC-3′) in the pDNA sequence allowed its interaction with p50 and p65 subunits of NFκB and improved its nuclear import via α- and β-importins [10,11]. The p65 subunit of NFκB that bears the NLS (nuclear localization signal) was demonstrated to directly interact in the neuronal cells with the dynein two intermediate chains (ICs) of 74 KDa (IC74) as well as dynactin p150 [12]. The p50–p65 heterodimers would travel on the MTs through their interaction with dynein.

Due to their size, viruses encounter the same mobility problems as naked pDNA within the cell, but most of them exploit dynein for their propagation along the MTs towards the cell nucleus. Thus, virus-mimicking strategies attempt to exploit dynein to improve pDNA transport to the nucleus for efficient non-viral gene transfer.

In this review, an overview on MTs, dynein, dynein/virus interaction feature is presented followed by a summary of non-viral transfection results obtained by exploitation of LC8 and TCTEL1 dynein light chain association sequence (DLC-AS). Then, the transfection efficiency of polyplexes and lipoplexes containing pDNA linked to a peptide derived from the adenoviral protein E3-14.7K is reported.

## Microtubles and dynein

MTs form hollow tubular structures with a diameter of 25 nm. Their wall is made up of, on an average, 13 protofilaments of tubulin which are laterally joined together. Each protofilament consists of a helical chain of heterodimers of α and β tubulin. Both are capable of binding GTP but only GTP bound to subunit β is exchangeable and hydrolysable to GDP and inorganic phosphate (Pi) [13]. MTs are polarized structures, each end can be considered as a pole. The negative pole has only α-subunits whereas the positive pole contains only β-subunits [14]. The positive ends are usually localized in the cellular periphery; the negative ends are grouped near the nucleus at the MT organizing centre (MTOC). MTs are very dynamic structures that are continually reorganizing. Elongation and shortening phases alternate with transition phases called disasters and rescues [15–17]. This dynamic instability occurs mainly at the positive (+) end; the negative (−) end being mostly sequestered at the centrosome. MT dynamics, including the balance between polymerization and depolymerization, are regulated by two groups of MT associated proteins (MAPs). Some MAPs are stabilizers. The best known example is the protein τ, which interacts with MTs and promotes their polymerization. Other MAPs such as stathmin destabilizes MTs by promoting their depolymerization. The MT network is strongly dynamic and plays a crucial role in a large number of critical functions such as the cellular architecture, cell division, vesicular transport, organelle and chromosome movement, and cell migration. Dynein and kinesin are two families of motor protein complexes that provide contrary movements along MTs. They actively guide organelles and vesicles through the cytoplasm. The heads of these complexes interact with MTs, while the tail interacts with vesicles and organelles. Kinesins (with the exception of kinesin 14) move towards the (+) end of MTs to the cellular periphery. Dynein moves towards the (−) end in the direction of the cell nucleus. All dynein isoforms are organized similarly and are macromolecular complexes of heavy, intermediate and light chains [18]. Within this complex, the heavy chain in two copies is responsible for the motor activity. The cytoplasmic dynein 1 is the major molecular motor for the displacement of organelles and vesicles (called cargos) in the cytoplasm of most eukaryotic cells [19,20].

**Figure 2:**
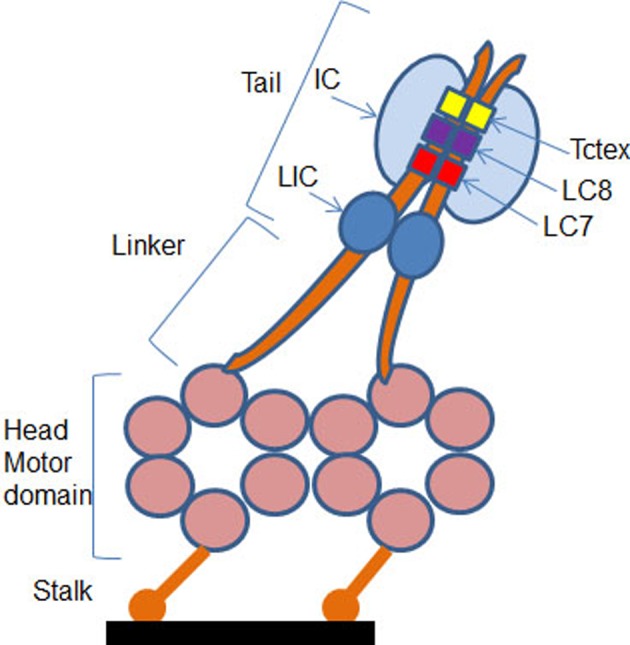
Schematic representation of the subunit composition of dynein The motor containing cytoplasmic DHCs are shown in orange and associated intermediate and light chains in shades of blue. The motor domain is composed of six AAA ATPase domains arranged in a hexameric ring from which a MT binding stalk projects. The N-terminal tail of the heavy chain mediates its dimerization and contains the binding sites for two ICs and two LICs. The two ICs also interact with three pairs of light chains: Tctex, LC7 and LC8 (adapted from [24]).

Dynein is a huge protein complex of 1.2 MDa molecular weight comprising two globular heads with a common base through thin stems that are the two heavy chains of the dynein (DHC, dynein heavy chain) of 530 kDa – containing the hydrolysis site of the ATP – interacting with MTs (Figure 2). Two IC74 and four light ICs (LICs) interact directly with the DHCs [21]. Three different light chain homodimers interact with ICs: TCTEX-1 (DYNLT), LC8 (DYNLL) and LC7 (DYNLRB) [22–24]. Dynein interacts directly with MTs via the MT binding domain (MTBD) located at the end of a long and thin stem of 10–15 nm at the AAA4 domain of the DHC [25]. The interaction interface of MTBD with MTs consists of a group of helix H1, H3 and H6 [26,27]. Dynein interacts via dynactin – a dynein activator molecule – with certain cellular cargos. The dynactin complex of 1 MDa molecular mass is composed of 11 different subunits. Among them is the p150 subunit which interacts with MTs and IC of the motor complex [28–31]. The presence of dynactin alongside dynein is essential for the total activity of the motor protein [32–36]. Like dynactin, the LC8 and TCTEX-1 homodimers allow interaction of the dynein complex with cargo vesicles. The homodimers of light chains of the dynein thus interact with the rest of the dynein complex (IC74) on one hand, and with the cargos on the other hand. Although different in their sequences, LC8 and TCTEX-1 have very similar tertiary structures comprising two α-helix followed by five β-leaflets, but their interactions with the cargos are different. The LC8 homodimer interacts on both IC74 at the level of the grooves between the two monomers which are also the sites for the interaction with the cargo molecules. As an adapter for cargo transportation, this homodimer must therefore replace one of the two ICs. Interaction with these sites is governed by a consensus peptide sequence which is of two types: KXTQTX or XG (I/V) QVD, where glutamine (Q) has a central position and interacts with the N-terminal part of the second α-helix of LC8, while the rest of the peptide is located in furrow between monomers [37].

Like LC8, a peptide motif of 19 amino acids has been identified in IC74 to bind to TCTEX-1 [38,39]. Within this peptide, a consensus sequence (R/K) (R/K) XX (R/K) such as RRLNK is found in many proteins interacting with TCTEX-1 [39]. The latter can therefore interact with cargos and IC74 either by the same side or by two opposite sides of the structure guaranteeing less competition (Figure 2). In addition to IC74, TCTEX-1 interacts with many other cellular partners such as rhodopsin [40], Doc-2 [41], polio virus CD155 receptor [42], the parathyroid hormone receptor [43], the capsid VP26 protein Herpes simplex [44] and fourteen seven K-interacting protein-1 (FIP-1) [45]. The highly conserved peptide sequences known to interact with the dynein light chains are termed as DLC-AS [46]. Compared with kinesin, the dynamics of dynein on MTs is still poorly known. The motive force driving the dynein transport mechanism is generated by a change in the position of the binding domain bridging the tail with the AAA1 domain of the DHC which acts as a lever during the cATP hydrolysis [47–52]. This phenomenon is coupled with a modification of the affinity of MTBD with MTs. The coupling of these two phenomena is the key to the dynein movement and requires communication between the head and the stem [53]. Dynein is said to ‘walk’ on MTs, so when one of the MTBDs is free, the second one is interacting with MTs. The dynein movement in the cells can reach ~1 μm/s depending on the forces applied [54].

## Dynein and viruses

Some viruses use dynein to move to the MTOC during the infection process. This active transport along MTs is critical for an effective viral infection process [55,56]. Depending on the virus type, recruitment of dynein engages different processes and partners (Table 1) [24,57]. For the movement of adenoviruses, hexon from the capsid subunit interacts directly with the IC and the LIC1 of dynein [58]. These interactions can take place providing that the hexon has been subjected to an acidic pH. Thus only viruses that have been translocated by endocytosis vesicles are capable of recruiting dynein.

**Table 1:** Viral proteins involved in the interaction of viruses with dynein

Virus	Viral protein	Dynein
Herpes simplex	UL34	DYNC1L1a
	UL9	DYNLL1 (LC8)
	UL35	DYNLT1 and 3 (TCTEX-1)
Herpes 7	UL19	DYNLL1 (LC8)
African swine fever	P54	DYNLL1 (LC8)
Mokola	Phosphoprotein P	DYNLL1 (LC8)
Rabies	Phosphoprotein P	DYNLL1 (LC8)
Papillomavirus	L2 capsid protein	DYNLT1 and 3 (TCTEX-1)
Adenovirus	capsid hexon	DYNC1LI1 and 2
Borna disease	G protein	DYNLRB1
Poliovirus	CD155 receptor	DYNLT1
HIV	Integrase	DYNLL1
Mason-Pfizer monkey	Viral matrix	DYNLT1
Ebola	Phosphoprotein	DYNLL1 (LC8)

Adapted from [57].

Dynactin is not involved in the recruitment of dynein by adenoviruses. For Herpes-like virus, the interaction with dynein takes place with capsid proteins and skin proteins around it. UL9, UL34, UL35 proteins interact directly with different components of the motor complex [44,59]. UL9 has a consensus sequence (746-KSTQT-750) for binding to DYNLL1, also called as LC8 [59]. UL35 of the VP26 capsid binds directly to TCTEX-1 and TCTEX-3. Among the various subunits constituting dynein, light chains are mainly targeted by viruses, and in particular LC8. Viruses recruit dynein via DLC-ASs of LC8 (Table 2) [59–61]. The first direct demonstration showing that DLC-ASs can facilitate an MT-dependent nuclear accumulation of a cargo protein was obtained with the rabies phosphoprotein (RPP). When RPP-139-174 as well as RPP-139-151 containing DLC-AS (DKSTQT) of LC8 was fused with GFP, they interacted with MTs and mediated GFP nuclear accumulation [46].

**Table 2:** Viral proteins interacting with LC8 through a DLC-AS

Protein	Virus	DLC-AS	Reference
Protein P	Rabies	RSSEEDKS**TQT**T	[60]
Protein P	Mokola	KSTEDKS**TQT**P	[60]
UL19	Human Herpes 7	TILSRS**TQT**G	[59]
		LGHFTRS**TQT**S	
UL9	Human Herpes 1	GVQMAKS**TQT**F	[59]
VP35	Ebola	PKTRNSQ**TQT**D	[61]
ADE41	Adenovirus	CITLVKS**TQT**V	[59]
P54	African swine fever	VTTQNTAS**QT**M	[59]

## Dynein and non-viral gene transfer

The idea of exploiting dynein to improve the cytosolic transport of pDNA in the context of non-viral gene transfer is fairly recent (Table 3). A positive effect has been reported when the peptide (KSSQDKSTQTTGD) from RPP-139-174 containing the DKSTQT motif was grafted via a disulphide bond directly on stearoyl–CH_2_R_4_H_2_C/pDNA complexes [62]. While the presence of the peptide reduced the uptake by the cells of the DLC-AS/pDNA complexes, the transgene expression was significantly improved. Another positive effect was reported when the hexon from adenoviral capsid was covalently conjugated to PEI of 800 kDa [63]. The resulting polyplexes increased by ten times the transgene expression into HepG2 cells compared with PEI/DNA complexes, but the interaction of polyplexes with MTs via hexon was not proved. Toledo et al. [64], developed a recombinant protein called LD4 made of a DNA-binding sequence (DNAb4: WRRRGFGRRR) fused to the N-terminus of the recombinant human dynein light chain LC8. pDNA/LD4 complexes had an enhanced capacity to interact and condense pDNA, and transfected HeLa cells. Despite transfection inhibition in the presence of nocodazole suggesting the involvement of MTs, there was no proof for a direct and specific interaction of pDNA/LD4 with dynein. Therefore, improved transfections have been obtained via targeting LC8. However, exogenous cargos bearing LC8 DLC-AS peptide have a low probability of meeting its target on the motor complex due to a strong competition with free endogenous LC8. Indeed, only 12% of the intracellular LC8 is integrated into the motor complex [65] thus the interaction sites of the LC8 DLC-AS are naturally occupied by IC74 [66]. Contrary to LC8, the TCTEX-1 dimer interacts with IC74 and cargos by two different mechanisms within the dynein complex in the absence of competition [67]. TCTEX-1 is almost exclusively within the dynein complex [68]. TCTEX-1 targeting sequences would be more potent candidates for DNA interaction with dynein. These sequences would thus guarantee a better specificity and would be less in competition. Favaro et al. [69], have developed a T-Rp3 recombinant protein containing the recombinant human dynein light chain Rp3 fused to its N-terminal end with a DNA-binding domain (DNAb4: WRRRGFGRRR) and to its C-terminal end with the membrane active peptide TAT (YGRKKRRQRRR). The human Rp3 is a member of the Tctex dynein light chain family and is associated with TCTEX-1 [21]. Transfection of HeLa cells with pDNA/T-Rp3 complexes was highly dependent on MT polarization. Luciferase expression was reduced by 92% in the presence of nocodazole while the transfection efficiency with protamine and Lipofectamine was only decreased by 56 and 41% respectively. Although this was not a proof for a direct and specific interaction of DNA complexes with dynein, these results suggested that efficacy of pDNA/T-Rp3 complexes strongly rely on active transport along MTs. The last 40 amino acids of L2 protein of the human papillomavirus capsid protein exhibiting the consensus sequence (R/K) (R/K) XX (R/K) have been identified to interact with TCTEX-1 [70]. It is noticeable that all those attempts to increase the transfection efficiency by exploiting migration on MTs were realized by coupling DLC-AS on to the cationic carrier – either a polymer or a recombinant fusion protein – but not directly on the pDNA. Therfore, in case of dissociation of DNA complexes in the cytosol, pDNA will not be able to dock on MTs. To date, the only study on pDNA transport on MTs with a DLC-AS covalently linked to pDNA was reported by us by using a peptide derived from the E3-14.7K protein [71].

**Table 3:** Exploitation of DLC-ASs for non-viral gene transfer

Protein	DLC-AS ligand	Linkage	DLC	Reference
Rabies P-protein	KSSQDKSTQTTGD	Lipopolymer	LC8	[62]
Adenoviral capsid	Hexon	PEI	LC8	[63]
LC8	LD4: LC8-DNAb4	Peptide	Dynein	[64]
Rp3	T-Rp3: DNAb4-Rp3-TAT	Peptide	TCTEX-1	[69]
E3 14.7K adenovirus	VVMVGEKPITITQHSVETEG	pDNA	TCTEX-1	[71]

## E3-14.7K protein

**Figure 3:**
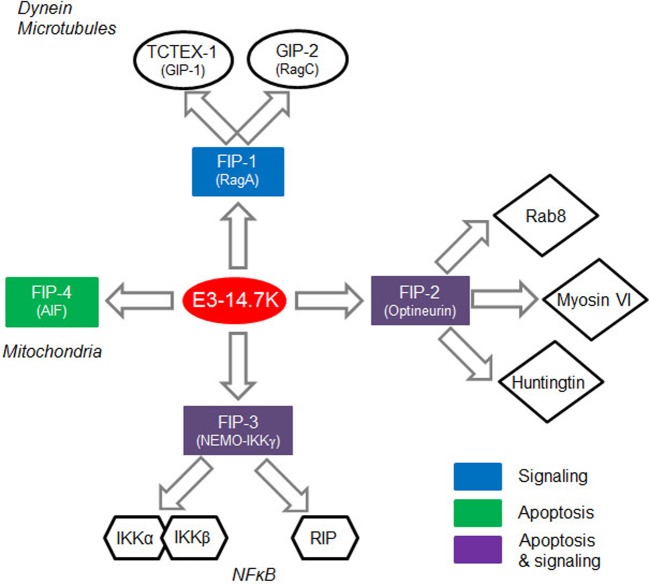
The interaction of E3-14.7K with FIPs FIP-1 plays a role in cell signalling through its involvement in intracellular trafficking of macromolecules. FIP-2 is involved in the TNF-α signalling pathway. FIP-3 is a regulatory protein of the NFκB pathway. FIP-4 is a proapoptotic molecule located in the mitochondrial intermembrane space (adapted from [74]).

The E3-14.7K early protein of human adenoviruses interacts with the TCTEL1 dynein light chain (TCTEX-1 homologue) via FIP-1, a cytosolic protein [45]. Viral E3 transcription unit encodes seven proteins named as E3-12.5K, E3-6.7K, E3-gp19K, E3-11.6K, E3-14.5K and E3-14.7K. As a rule, E3 proteins attack cell defence mechanisms such as antigen presentation, apoptosis and inflammatory response. E3-14.7K is mainly known to protect infected cells from TNF-α-induced cell death and to inhibit the inflammatory response. This protein is expressed by many adenoviral serotypes and its sequence is highly conserved [72]. Unlike other E3 proteins, E3-14.7K does not contain a transmembrane domain and is located both in the nucleus and the cytoplasm [73]. E3-14.7K interacts with four cellular proteins called FIPs (Figure 3) [74]. FIP-1, a small GTPase also known as RagA or RRag for Ras-related GTP-binding protein A, is a functional human homologue of *Saccharomyces cerevisiae* Gtr1p16. FIP-1 interacts with RagC and RagD17, and also with NOP132 nucleolar protein18. FIP-2, also known as NRP11 (NFκB essential modulator (NEMO) related protein) or optineurin12, is involved in the TNF-α signaling pathway. As a crucial subunit of the IKK complex, FIP-3 (NEMO or IKKγ) is a key regulator of the NFκB pathway. FIP-4 or AIF (apoptosis-inducing factor) is a mitochondrial protein which translocates into the nucleus in response to apoptotic stimuli but the significance of its interaction with E3-14.7K is still unknown. We recently identified a 20-amino acid peptide called P79-98 containing residues 79–98 from the amino acids sequence of E3-14.7K that specifically interacts with FIP-1 and we reported that when linked to a pDNA, it mediated interaction of pDNA with MTs and dramatically enhanced polyplexes transfection [71].

## E3-14.7K interacts with TCTEL1 via FIP-1 to form a MT interacting complex

**Figure 4:**
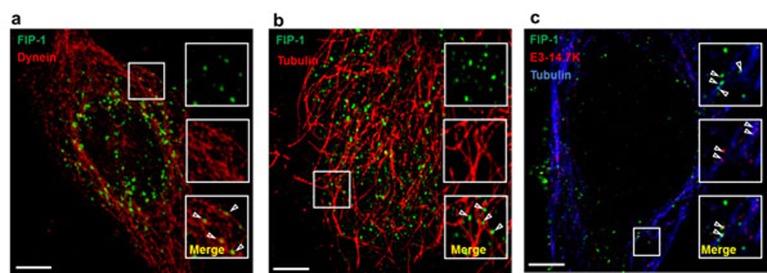
E3-14.7K/FIP-1 and MT interacting complex (**a**) Colocalization of FIP-1-eGFP (green) with cytoplasmic dynein (red): HeLa cells were transfected with a pDNA encoding FIP-1-eGFP (pFIP-1-eGFP). Two days after transfection, cells were treated with 1 mM DSP for 10 min in HBSS, pH 7.2 before permeabilization with digitonin. Cells were fixed and cytoplasmic dynein was stained with antidynein MAb revealed with Cy3 anti-mouse antibodies. (**b**) Colocalization of FIP-1-eGFP (green) with MTs (red) in HeLa cells: HeLa cells were transfected with pFIP-1-eGFP. Two days after transfection, cells were treated with 1 mM DSP for 10 min in HBSS pH 7.2 before permeabilization with digitonin. Cells were fixed and MTs were stained with anti-α-tubulin MAb revealed with Cy3 secondary anti-mouse antibodies (red). (**c**) Triple colocalization between FIP-1-eGFP (green), E3-14.7K-Tomato (red) and MTs (blue). HeLa cells were cotransfected with pFIP-1-eGFP and a plasmid encoding E3-14.7K-Tomato (pE3-14.7K-Tomato). Two days post-transfection, cells were treated with 1 mM DSP for 10 min in HBSS pH 7.2. Cells were then permeabilized, fixed and MTs were stained with anti-α-tubulin MAb revealed with Cy5-labelled secondary anti-mouse antibodies (blue). Colocalization experiments were performed by confocal laser scanning microscopy using a Zeiss Axiovert 200M microscope coupled with a Zeiss LSM 510 scanning device. Boxes on the right correspond to the enlarged ROI (square). Scale bar: 5 µm. pE3-14.7K-Tomato was pDNA encoding E3-14.7K fused with td-Tomato under the control of the CMV promoter. pFIP-1-eGFP was a homemade pDNA constructed from pcDNA-T7-FIP-1 (kindly given by Prof M.S. Horwitz, Albert Einstein College of Medicine, New York, NY, U.S.A.) [45] and peGFP-N3 from Clontech (Mountair View, CA, U.S.A.) encoding eGFP driven by CMV promoter.

**Figure 5:**
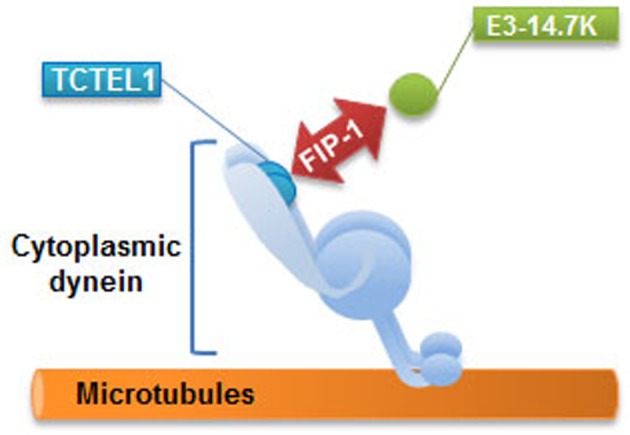
Scheme of E3-14.7K interaction with TCTEL1 via FIP-1 to form a MT interacting complex

Since FIP-1 interacts with the GIP1 (GTPase interacting protein-1)/TCTEL1 complex [75], the interaction between FIP-1, E3-14.7K and MTs was evaluated in HeLa cells upon co-transfection with a pDNA encoding for FIP-1 fused with eGFP and a pDNA encoding for E3-14.7K fused with td-Tomato. For a clear intracellular localization of these fluorescent proteins in interaction, the cells were treated first with 3,3′-Dithiodipropionic acid di(N-hydroxysuccinimide ester) (DSP) to cross-link proteins that were in close contact and then, they were gently permeabilized with digitonin in order to wash out soluble proteins that did not interact with any partners in the cytoplasm. In a representative image shown in Figure 4a, FIP-1 (green spots) is found close to MTs (red filaments). Interestingly, the distribution of the green spots were organized as a fibrillar network. In Figure 4b, fluorescent spots corresponding to FIP1-eGFP and E3-14.7K-Tomato were observed in the centre of the cell. Moreover, FIP1-eGFP (green spots) was also aligned as above and some of them co-localized with E3-14.7K-Tomato in the perinuclear area as indicated by the yellow foci. Of note, the position of eGFP tag at N- or C-terminus of FIP-1 had no influence on their subcellular localization (results not shown). Triple colocalization experiments were performed on cells co-transfected with pFIP-1-eGFP (green) and pE3-14.7K-Tomato (red) and MTs immunostained and revealed with a Cy5-tagged secondary antibody (blue). In Figure 4c, FIP-1-eGFP was close to MTs (blue). FIP-1-eGFP and E3-14.7K-Tomato were clearly colocalized (see enlarged area) and aligned along MTs following the interacting scheme shown in Figure 5.

## P79-98 peptide of E3-14.7K responsible of E3-14.7K/FIP-1 interaction mediates binding of cargo on to MTs

**Figure 6:**
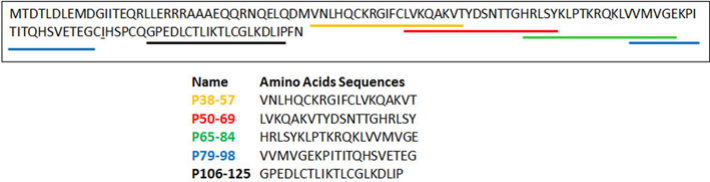
List of the selected peptides covering amino acids from position 38 to position 125 of E3-14.7K C-terminus

**Figure 7:**
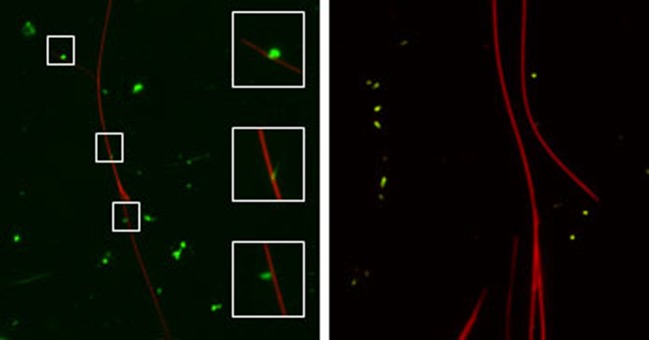
P79-98 peptide binding to isolated MTs X-rhodamine-labelled MTs (red) were polymerized *in vitro* and incubated with Qdot streptavidin (green) conjugate 545 labelled with biotinylated P79-98 peptide (bio-P79-98: VVMVGEKPITITQHSVETEG-Ttdsbiotin; left panel) or biotinylated P38-57 peptide (bio-P38-57: VNLHQCKRGIFCLVKQAKVTTtds-biotin; right panel) in HeLa cells cytosolic extracts supplemented with 10 mM ATP [71].

**Figure 8:**
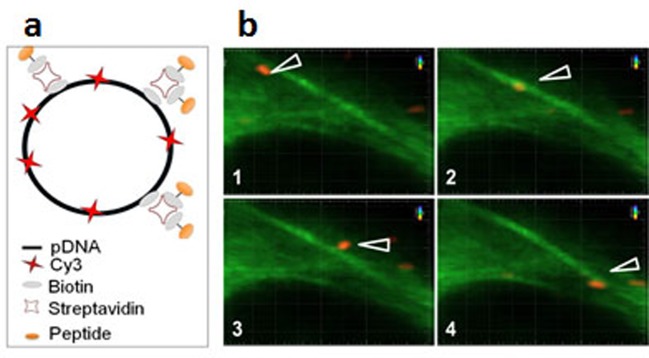
P79-98/pDNA moving on MTs (**a**) Scheme of assembly of biotinylated (bio) and Cy3-pDNA with biotinylated P79-98 peptide loaded streptavidin (STR). (**b**) P79-98 allows intracellular movement of pDNA on MTs. HeLa cells stably expressing eGFP-α-tubulin were transfected for 90 min with P79-98/Cy3-pDNA/His-lPEI complexes. Panels (1–4) show a representative time lapse acquisition (interval between each frame: 10 s) performed by videomicroscopy.

The determinants of E3-14.7K for recognition of FIP-1 are retained within residues 31–128 of its C-terminus fragment [76]. To identify the amino acid sequence involved in this interaction, five overlapping peptides of 20 amino acids length covering this 97-amino acid sequence were selected (Figure 6). When binding assay was performed by bioluminescence resonance energy transfer (BRET), only the P79-98 peptide was found to inhibit E3-14.7K/FIP-1 interaction [71]. To evidence that P79-98 can mediate the binding of a cargo to MTs, the biotinylated P79-98 peptide (P79-98-bio) was linked to Qdot 545 streptavidin (P79-98-Qdot) (Figure 7). The binding assay was performed by incubating P79-98-Qdot with isolated polymerized X-rhodamine MTs in cytosolic extract of HeLa cells supplemented with 10 mM ATP as described [71]. Fluorescence microscopy showed that P79-98-Qdot bound to MTs which was not the case in the control corresponding to the P38-57 peptide linked to Qdot 545 streptavidin (P38-57-Qdot) (Figure 7). Biotinylated P79-98 peptide has been then grafted on pDNA, thanks to streptavidin as described in Figure 8a and [71]. This scaffold has been used to form P79-98/Cy3-pDNA/His-lPEI polyplexes and the transfection was carried out in HeLa cells stably expressing eGFP-tubulin. A clear intracellular movement of red spot along MTs corresponding to pDNA particles was observed by videomicroscopy (Figure 8b, panels 1–4).

## Impact of P79-98 on the transfection efficiency of polyplexes and lipoplexes

**Figure 9:**
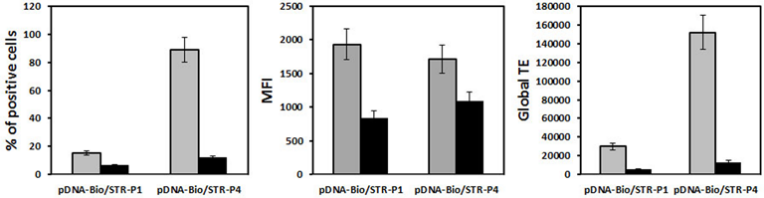
MT dependence HeLa cells were transfected with 2.5-µg pCMV-eGFP (5130 bp) encoding Yellow Fish enhanced GFP gene under the control of the CMV promoter. pDNA was biotinylated (pDNA-bio) (DNA/biotin molar ratio of 3) and associated either with bio-P79-98 (P4) or bio-P38-57 (P1) via streptavidin as described in Figure 8a. The equipped plasmid was then complexed with His–lPEI (DNA/polymer weight ratio of 1/6). The transfection was performed for 4 h at 37°C in the absence (grey bar) or the presence (black bar) of 33 µM nocodazole. Then the cells were washed and incubated for 48 h in fresh medium in the absence of nocodazole and any polyplexes. The fluorescence of cells was measured after 48 h transfection by flow cytometry. % stands for the percentage of transfected cells, MFI for the mean of the fluorescence intensity of the transfected cells and global transfection efficiency (TE) for the mathematical product of the MFI of cells and the percentage of fluorescent cells.

**Figure 10:**
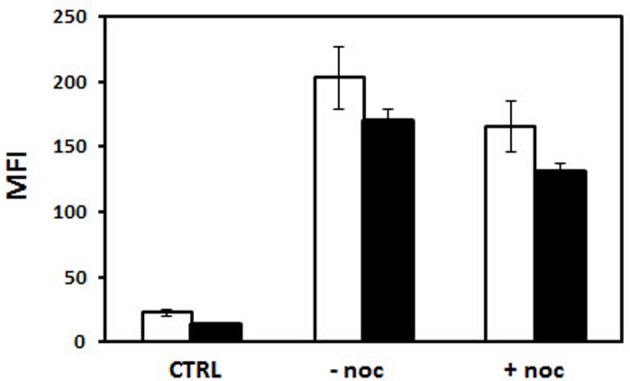
Uptake of polyplexes in the presence of nocodazole HeLa cells were incubated for 4 h in the absence and the presence of 2.5 µg pDNA. Fluorescein-labelled pDNA was biotinylated (pDNA-bio) (DNA/biotin molar ratio of 3) and associated either with bio-P79-98 (P4) or bio-P38-57 (P1) via streptavidin as described in Figure 8a. The equipped fluorescent plasmid was then complexed with His–lPEI (DNA/polymer weight ratio of 1/6). The incubation was performed for 4 h at 37°C in the absence or the presence of 33 µM nocodazole. Then the cells were washed and the fluorescence of cells was measured by flow cytometry in the absence (white bar) and in the presence (black bar) of Trypan Blue.

When HeLa cells were transfected with His–lPEI polyplexes made with bio-peGFP conjugated with P79-98-STR, the number of eGFP transfected cells was eight-fold higher than transfection performed with bio-peGFP conjugated with P38-57-STR that did not interact with FIP-1 (Figure 9). Approximately 90% of the cells expressed eGFP when transfection was performed with P79-98/peGFP compared with 15% with P38-57/peGFP. These results confirmed the specific involvement of P79-98 in the enhancement of the transfection efficiency with P79-98/peGFP polyplexes reported in [71]. It is worth noticing that the level of the gene expression (MFI) was similar whatever the peptide used meaning that the improvement of the cytosolic migration of pDNA on MTs resulted in the enhancement of the number of transfected cells rather than in the gene expression level. The benefit of P78-98 was due to its capacity to promote pDNA binding and its migration on MTs. Indeed, in the presence of nocodazole that inhibits MTs depolymerization, the number of transfected cells drastically dropped from 90 to 10% (Figure 9). Comparatively, it was less decreased in the case of transfection performed with P38-57/peGFP polyplexes. The nocodazole effect was lowering the MFI level indicating its impact on the gene expression. Note that this was not due to a reduction in the amount of internalized polyplexes in the presence of nocodazole. Indeed, the MFI values were similar in the absence and the presence of nocodazole (Figure 10). Thus, the presence of P79-98 linked to the pDNA promoted its accumulation near the nuclear envelope thanks to MT transport and was of benefit for its nuclear import in a larger number of cells.

**Figure 11:**
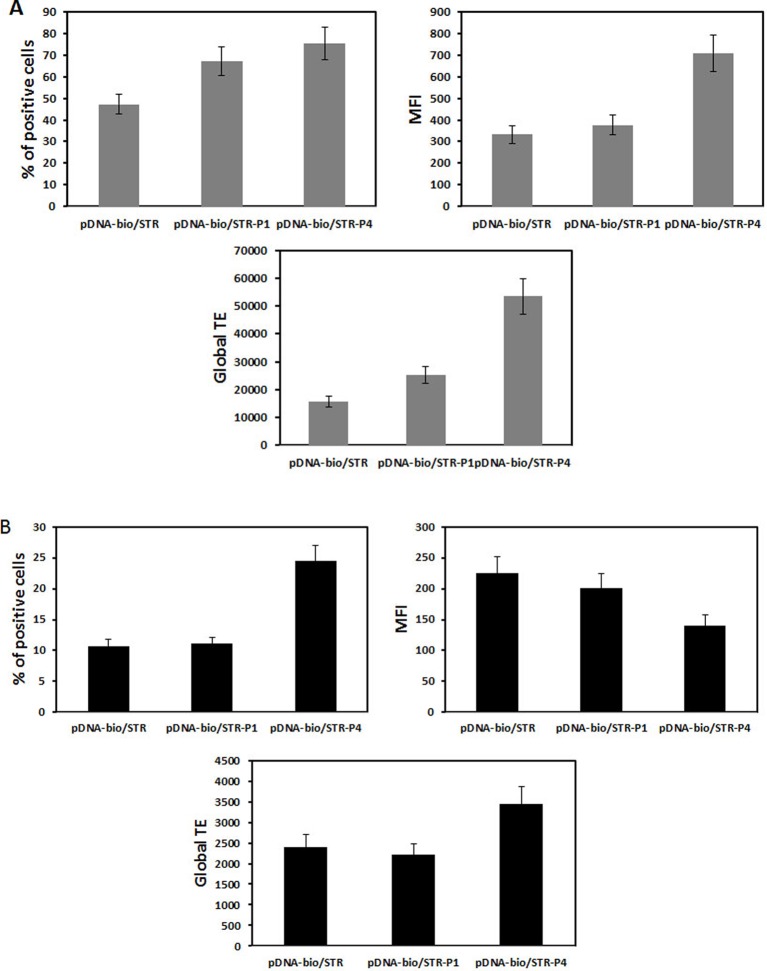
Transfection of mouse myoblasts and human bronchial epithelial cells. P79-98 increases the transfection efficiency in (**A**) C2C12 and (**B**) 16HBE14o- cells. Cells were transfected with 2.5 µg pCMV-eGFP as described in Figure 9. The fluorescence of cells was measured after 48-h transfection by flow cytometry and data were given as the percentage of transfected cells (% of positive cells), the mean of the fluorescence intensity (MFI) of the transfected cells and the global transfection efficiency (TE) (i.e. the mathematical product of the MFI of cells and the percentage of fluorescent cells).

**Figure 12:**
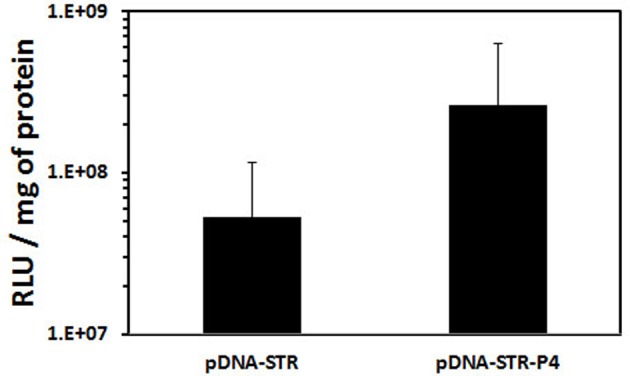
Liver transfection efficiency of pDNA-P79-98 Hydrodynamic injection of 15 µg of biotinylated p3NF-CMV-luc-3NF conjugated with streptavidin (pDNA-STR) or biotinylated p3NF-CMV-luc-3NF conjugated bio-P79-98 linked to streptavidin (pDNA-P4) was performed in the tail vein of CD1-Swiss mice (six mice each). Mice were injected in less than 8 s via the tail vein with polyplexes in 2.5 ml of isotonic NaCl + 5% glucose. Three days post-injection, mice were anaesthetized by isofluran inhalation then killed by cervical dislocation. The liver was removed, crushed in passive lysis buffer (Promega, Charbonnières Les Bains, France) using the gentle MACS dissociator (Miltenyi Biotech, Paris, France). After centrifugation of homogenates, the luciferase activity was measured and expressed as RLU per mg of proteins. p3NF-CMVLuc-3NF (5556 bp) encoding the firefly luciferase cassette under the control of a CMV promoter and containing 3NF sequences recognized by NFκB was constructed from p3NF-Luc-3NF [78] by replacing pTAL promoter by CMV promoter.

When transfection with polyplexes made either with peGFP, peGFP-linked P38-57 or peGFP linked to P78-98 was performed on the mouse myoblast cells (C2C12 cells; CRL1772; ATCC, Rockville, MD, U.S.A.) and the human bronchial epithelial cells (16HBE14o-), the global transfection efficacy depended on the cell lines; it was greater in C2C12 cells than in 16HBE14o- cells. But in both the cell lines, the number of transfected cells was increased. It was 100 and 60% higher with peGFP linked to P78-98 than with peGFP linked P38-57 in C2C12 cells and 16HBE14o- cells respectively (Figure 11). Even though the eGFP expression in 16HBE14o- cells was lower, the number of transfected cells (25%) was 2.2-fold higher with peGFP linked to P79-98 than with peGFP-linked P38-57 (Figure 11). The impact of the linkage of P78-98 on pDNA on transfection was also tested *in vivo*. For this purpose, hydrodynamic injection consisting of rapid administration of a large volume of pDNA was used. When performed via the tail vein of mice, it is highly efficient for liver transfection [77,78]. As shown in Figure 12, the luciferase activity measured in the liver of mice 3 days after the injection upon hydrodynamic administration of naked pDNA-P79-98 was five-fold higher than that recorded with pDNA-STR. Thus, the equipment of pDNA with the P79-98 peptide was also benefitting in *in vivo* transfection.

**Figure 13:**
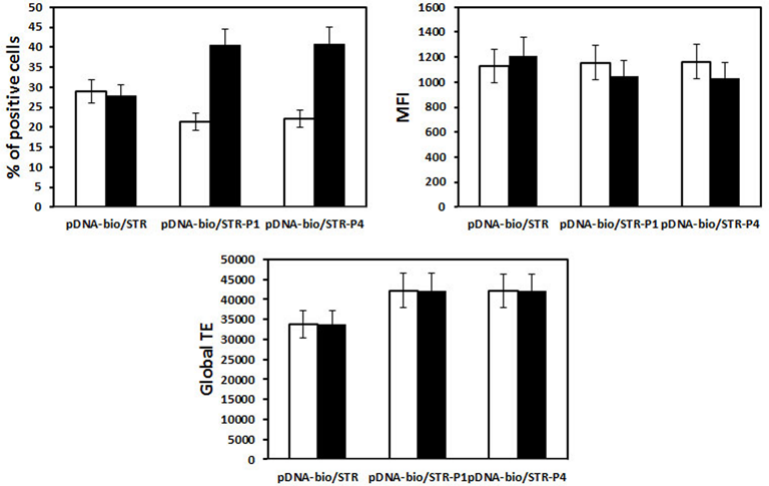
Influence of P79-98 on the transfection efficiency of HeLa by lipoplexes HeLa cells were transfected with 2.5-µg pCMV-eGFP. The plasmid was biotinylated (pDNA-bio) (biotin/DNA molar ratio of 3) and associated via streptavidin (STR) either with bio-P79-98 (P4) or bio-P38-57 (P1) as described in Figure 8a. The equipped plasmid was then complexed either with (white bar) KLN25/MM27 (DNA/lipid weight ratio of 1/2) or (black bar) Lipofectamine cationic liposomes. The fluorescence of cells was measured after 48-h transfection by flow cytometry and data were given as the percentage of transfected cells (% of positive cells), the mean of the fluorescence intensity (MFI) of the transfected cells and the global transfection efficiency (global TE) (i.e. the mathematical product of the MFI of cells and the percentage of fluorescent cells).

**Figure 14:**
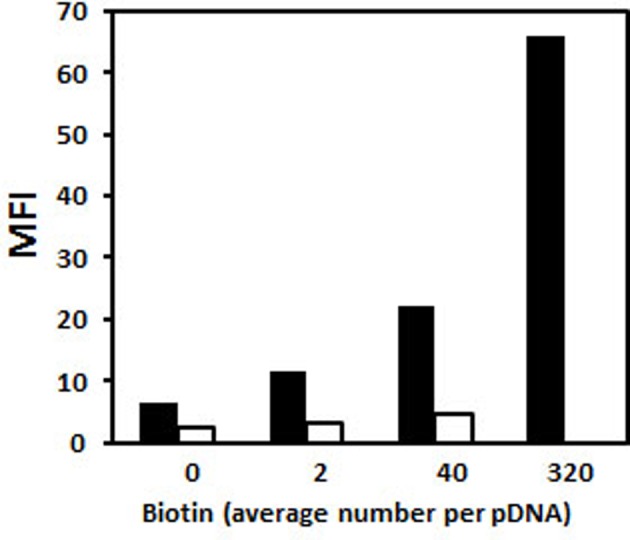
Biotin accessibility within polyplexes and lipoplexes pDNA (pCMV-eGFP) was substituted with various amounts of biotin residues. Polyplexes were formed with His–lPEI (black bar) at DNA/polymer weight ratio of 1/6. Lipoplexes were formed with KLN25/MM27 liposomes (white bar) at DNA/lipid weight ratio of 1/2. DNA complexes were mixed with fluorescein-labelled streptavidin and then the fluorescence intensity of polyplexes and lipoplexes was measured by flow cytometry.

The effect of P79-98 was also evaluated for transfection of HeLa cells with lipoplexes (cationic lipid/DNA complexes) made either with KLN25/MM27 cationic liposomes [79] or Lipofectamine. As shown in Figure 13, the number of transfected cells (~30%) and the level of the gene expression (MFI ~1100) was relatively good but no effect of P79-98 was observed. Thus, the impact of the P79-98 peptide on the transfection efficiency could depend on the type of vector (polymer compared with liposomes). These results raised several questions. First, is that the peptide linked to pDNA was accessible to FIP-1 in the absence of polyplexes and lipoplexes dissociation? To answer the question, we tested the binding of fluorescein-labelled streptavidin on to polyplexes and lipoplexes made with biotinylated pDNA by flow cytometry. As shown in Figure 14, streptavidin bound to His–lPEI polyplexes as a function of the number of biotin linked to pDNA whereas it failed in case of KLN25/MM27 lipoplexes whatever the number of biotin linked to pDNA. This means that biotinyl groups were hidden within those lipoplexes but not within HislPEI polyplexes. Assuming that results with biotin linked to pDNA can be translated to P79-98 linked to pDNA, these data suggested that P79-98 pDNA could bind to MTs in the cells even in the absence of dissociation of His–lPEI polyplexes. In the case of lipoplexes, the absence of P79-98 pDNA release from KLN25/MM27 or Lipofectamine in the cytosol or its rapid degradation before reaching MTs or during its migration on MTs after lipoplexes dissociation could explain the absence of transfection improvement. The intracellular mechanism leading to P78-98 pDNA *in cellulo* interaction with MTs and transfection improvement is not yet deciphered and requires more studies with polyplexes and lipoplexes. As pointed by the lipoplexes results, knowledge on the intracellular DNA complexes stability would be crucial. Other types of polymers and liposomes could be tested and it would be interesting to correlate the transfection results with the capacity of DNA complexes to release pDNA. In case of strong stability, the linkage of P79-98 to polymer, polyplexes, liposomes or lipoplexes could be evaluated even though this could affect the shape of DNA complexes, their interaction with the cell surface and physiological environments such as serum proteins, their internalization process and intracellular trafficking. However, uptake process did not seem to have influence since KLN25/MM27 and Lipofectamine lipoplexes were internalized via caveolae and clathrin-dependent endocytosis respectively [79].

## Conclusion

This review aims to summarize dynein feature and how dynein can be exploited to improve pDNA mobility in the cytosol in order to facilitate its accumulation near the nuclear envelope and more precisely near the nuclear pores. Although the use of LC8 or TCTEL1 DLC-AS peptides is obvious, modest transfection benefits were reported with lipoplexes or polyplexes when they were linked to cationic polymers or lipids. But, pDNA will not be able to dock on MTs in case of DNA complexes dissociation. In contrast, the linkage of pDNA with the P79-98 peptide derived from E3 14.7K recognizing FIP-1 interacting with TCTEL1 showed a good interaction with MTs *in cellulo* and a high transfection improvement. A comparative transfection efficacy with pDNA coupled either to P79-98, LC8 or TCTEL1 DLC-AS deserves to be conducted even though the linkage of peptides to pDNA remains challenging.

## Abbreviations

DHC: dynein heavy chain
DLC-AS: dynein light chain association sequence
DYNC1: cytoplasmic dynein 1
DYNLRB: roadblock dynein light chain
DYNLT: dynein light chain of 13 kDa
FIP-1: fourteen seven K-interacting protein-1
IC: intermediate chain
His: histidine
IC74: intermediate chains of 74 kDa
LD4: dynein light chain domain 4
LIC: light intermediate chain
LC8: dynein light chain of 8 kDa
MAb: monoclonal antibody
MAP: microtubule associated protein
MT: microtubule
MTBD: microtubule binding domain
MTOC: microtubule organizing centre
NEMO: NFκB essential modulator
pDNA: plasmid DNA
PEI: polyethylenimine
ROI: region of interest
RPP: rabies phosphoprotein
TCTEX: T-complex testis-specific protein
